# Treatment and Management of Anaplastic Thyroid Carcinoma: Appraisal of Clinical Practice Guidelines

**DOI:** 10.1017/S0022215124001087

**Published:** 2025-01

**Authors:** Jonathan P. Kuriakose, Najm Khan, Neeraj V. Suresh, Emma De Ravin, Alvaro Moreira, Karthik Rajasekaran

**Affiliations:** 1 Robert Wood Johnson Medical School, Rutgers University, New Brunswick, NJ 08901, USA; 2 Department of Otorhinolaryngology, University of Pennsylvania, Philadelphia, PA, 19104 USA; 3 Perelman School of Medicine, University of Pennsylvania, Philadelphia, PA, 19104 USA; 4 Division of Neonatology, Department of Pediatrics, University of Texas, San Antonio, TX, 78229 USA

**Keywords:** thyroid, cancer, evidence-based medicine, treatment outcome, systematic review

## Abstract

**Objective:**

To appraise clinical practice guidelines for anaplastic thyroid carcinoma treatment and management using the Appraisal of Guidelines for Research and Evaluation II tool.

**Methods:**

A literature search was performed using MEDLINE/PubMed, Embase, Scopus, Cochrane, and Google Scholar. Four reviewers evaluated clinical practice guidelines utilising Appraisal of Guidelines for Research and Evaluation II, with domain scores requiring a threshold of greater than 60 per cent. Inter-reviewer agreement was evaluated using intraclass correlation coefficients.

**Results:**

Twelve clinical practice guidelines were evaluated after application of inclusion and exclusion criteria. There were two “high-”, four “average-”, and six “low-” quality clinical practice guidelines. The domains with the highest scores were “clarity of presentation” (69.44 ± 16.75) and “scope and purpose” (68.87 ± 20.88), while “applicability” (7.12 ± 6.17) and “rigor of development” (50.26 ± 20.77) had the lowest scores. Intraclass correlation coefficients showed a high level of inter-reviewer agreement (0.689–0.924; good–excellent).

**Conclusion:**

These results showcased wide variability in quality amongst guidelines for the treatment and management of anaplastic thyroid carcinoma. These findings necessitate greater standardisation among clinical practice guidelines and greater focus on the applicability of recommended practices.

## Introduction

Anaplastic thyroid carcinoma is a highly aggressive malignant tumour that occurs in 2–3 per cent of all thyroid gland neoplasms.^1^ It most often presents as a rapidly growing, firm, painful, fixed, anterior neck mass with compressive symptoms such as hoarseness and dysphagia. These masses are first evaluated with ultrasonography. If signs of malignancy such as hypoechogenicity, irregular margins, internal calcifications, or cervical lymph node involvement are seen, a fine needle aspiration (FNA) is performed. On cytology, anaplastic thyroid carcinoma shows focal clusters of atypical cells, mitotic figures, and in some cases have background necrosis and inflammatory cells. If signs of malignancy are noted in the FNA or if there remains high clinical suspicion for anaplastic thyroid carcinoma, a computed tomography (CT) scan and magnetic resonance imaging (MRI) are performed to identify the extent of local tumor invasion, lymph node metastases, and distant metastases. Unfortunately, patients often initially present with distant metastases and regional lymph nodes positive for carcinoma. As such, anaplastic thyroid carcinoma has a very poor prognosis with nearly 100 per cent mortality.

Treatment of anaplastic thyroid carcinoma is variable but most commonly involves debulking surgery, which is done to remove any tumour that is compressing the airway or at high risk of threatening airway patency.^2^ Due to the extent of invasion and high occurrence of metastases, patients with anaplastic thyroid carcinomas always receive adjuvant external beam radiation. If surgery is forgone, both radiation and chemotherapy are given. Due to the poor prognosis, palliative care expertise is often utilized to control pain and compressive symptoms, while also addressing psychosocial implications of the disease. If a patient refuses all treatment or if the anaplastic thyroid carcinoma is rapidly progressing despite treatment, hospice care is often provided to patients.

Since 2014, several medical organizations have developed clinical practice guidelines to identify best practices for anaplastic thyroid carcinoma treatment and management.^3–16^ Due to the low rate of occurrence yet high mortality, clinical practice guidelines vary greatly in regards to their treatment and management protocols for anaplastic thyroid carcinoma. Furthermore, there is little standardization in these clinical practice guidelines due to the multidisciplinary approach to the care of patients with anaplastic thyroid carcinoma as it can be treated by otolaryngologists and endocrine surgeons and require the assistance of radiologists, endocrinologists, palliative care, and hospice care.

With the high variability among the published clinical practice guidelines, our study aimed to appraise each of these clinical practice guidelines with the Appraisal of Guidelines for Research and Evaluation II tool.^17^ The Appraisal of Guidelines for Research and Evaluation II tool was developed to assess the quality and rigor of clinical practice guidelines using 23 standardized criteria assessing six quality domains: scope and purpose, stakeholder involvement, rigor of development, clarity of presentation, applicability, and editorial independence.

Several prior research studies have utilised the Appraisal of Guidelines for Research and Evaluation II instrument for clinical practice guideline appraisal, with a recent systematic review showing this tool to be the most effective tool for appraising clinical practice guideline quality.^18^ As such, we reviewed the literature to identify clinical practice guidelines for the treatment and management of anaplastic thyroid carcinoma and appraised them using the Appraisal of Guidelines for Research and Evaluation II tool with the goal of identifying and evaluating the heterogeneity among these clinical practice guidelines.

## Material and Methods

### Literature search & selection criteria

This review followed the 2020 Preferred Reporting Items for Systematic Review and Meta-Analysis (‘PRISMA’) guidelines.^19^ The published literature was queried for clinical practice guidelines addressing the treatment and management of anaplastic thyroid cancer. The search was performed utilizing the following databases from inception to 1 May 2022: Embase, MEDLINE/PubMed, Scopus, Cochrane, and Google Scholar. Search strategies were extensively tested in PubMed and reviewed by the research team before finalization. The final search strategy utilized was ((“anaplastic thyroid cancer” OR “anaplastic thyroid carcinoma”) AND (“guideline” OR “consensus” OR “recommendation” OR “clinical practice guideline”)).

**Figure 1.**
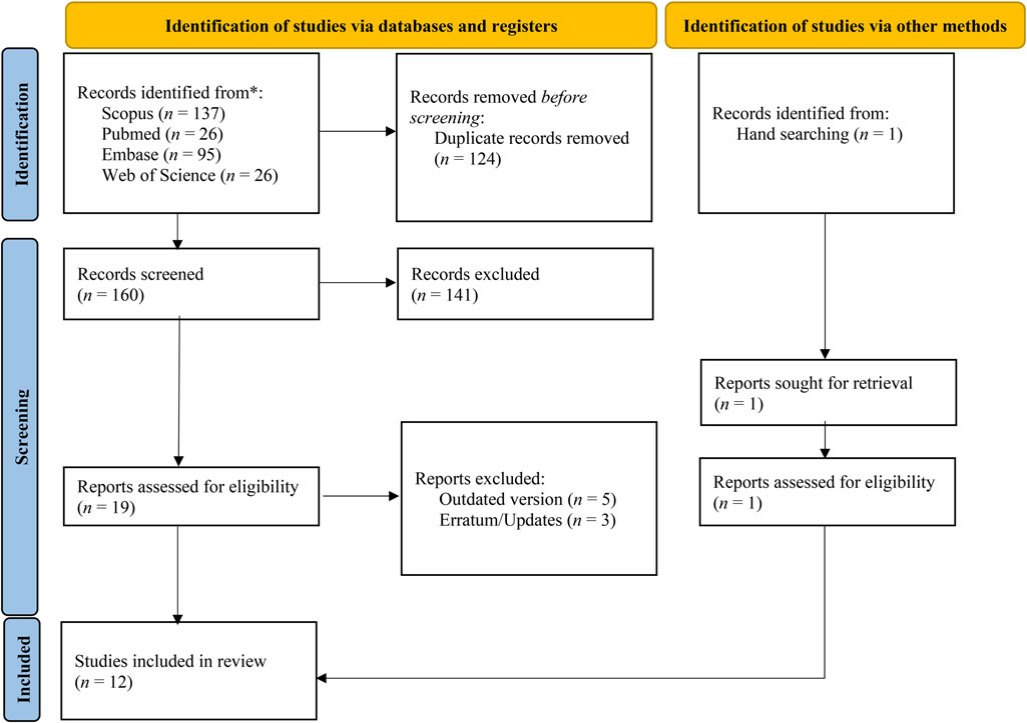
PRISMA Diagram of identified and included clinical practice guidelines.

References (*n* = 284) were obtained and managed using EndNote 20 (Clarivate Analytics, London, 2021); which produced 124 duplicates. The remaining 160 references were exported from EndNote into Rayyan (http://rayyan.qcri.org) for title and abstract screening by two independent reviewers (JK and NK). Nineteen references were selected for full-text screening and independently screened by the same two reviewers. Five reports had outdated guidelines and were excluded. Three reports were found to be errata for existing guidelines and were combined with their, respective, original reports. Hand searching identified one more report. The search methodology of identification, screening, and selection is displayed in Figure 1.

After duplicate removal, the initial review included the title and abstract screening. A study was included if it was a national or international guideline, consensus statement, or recommendation that reported the treatment and management of anaplastic cancer. If a society or group published multiple guidelines, we included the most updated report. Reports were excluded if they were: not published in a peer-reviewed journal, a review article, or not available in English. The utilization of Rayyan allowed the blinding of each reviewer's results and any discrepancies were resolved through a verbal discussion.

This study does not meet the definition of human-subjects research and is considered IRB exempt.

### Data extraction and management

After initial review and implementation of inclusion and exclusion criteria, the general characteristics of each guideline were gathered. A shared, Excel sheet was used to compile data based on the 23 items within the 6 domains of the Appraisal of Guidelines for Research and Evaluation II instrument. Each clinical practice guideline was reviewed to identify information such as the authoring organization, journal, year of publication, development method, development committee members, inclusion of patient organizations, target audience, steps for implementation of recommendations, number of references, and relevant funding.

### Quality appraisal

After completing the Appraisal of Guidelines for Research and Evaluation II online training, each guideline was independently reviewed using the Appraisal of Guidelines for Research and Evaluation II instrument by four of the authors (JK, NK, NS, ED). The Appraisal of Guidelines for Research and Evaluation II tool has six domains: (1) scope and purpose, (2) stakeholder involvement, (3) rigor of development, (4) clarity of presentation, (5) applicability, and (6) editorial independence. Each of these domains contain 2–7 specific items, with a total of 23 specific items in the entire Appraisal of Guidelines for Research and Evaluation II tool. Each item is scored 1–7 based on how well the guideline addressed the information specified in each item, with a score of 1 being “strongly disagree” and a score of 7 being “strongly agree.” After scoring of each item, a scaled percentage score was calculated for each domain utilizing a predetermined formula in the Appraisal of Guidelines for Research and Evaluation II manual: [(obtained score − minimum possible score)/(maximum possible score − minimum possible score)] × 100. A scaled percentage score of greater than or equal to 60 per cent was used as a threshold for gauging the quality of each clinical practice guideline. If a clinical practice guideline had five or more domains with a score achieving the threshold, it was a “high” quality clinical practice guideline. If 3–4 domains achieved threshold, it was an “average” quality clinical practice guideline. Fewer than 3 domains reaching threshold was defined as a “low” quality clinical practice guideline.^20^ Each clinical practice guideline received a final quality rating percentage, which was defined as the mean of the six scaled domain scores.

### Statistical analysis

Using Python 3.8 (www.python.org) and Pingouin API, the level of agreement and reliability among the four appraisers was determined using an intraclass correlation coefficient analysis with random-effects modelling. Intraclass correlation coefficient scores were stratified as poor (< 0.4), fair (0.40–0.59), good (0.60–0.74), and excellent (>0.74) based on prior literature.^21^ In addition, intraclass correlation coefficients were used to compare the level of generalizability of this study's appraisals with similar appraisers who had received Appraisal of Guidelines for Research and Evaluation II training.

## Results

### Literature search

Using the aforementioned keywords and phrases, a literature search was performed which identified 284 articles. After removal of duplicates and application of inclusion and exclusion criteria, 12 articles remained. The authors (JK, NK, NS) discussed any discrepancies regarding the clinical practice guidelines selected for appraisal. The step-by-step literature search methodology is shown in Figure 1.

### Guideline general characteristics

The included clinical practice guidelines and their general characteristics are shown in Table 1. All clinical practice guidelines were published between 2014 and 2021. Two of the clinical practice guidelines were international consensuses among various endocrinology organizations: one between the United States of America and Italy and the other among several European countries.^7,12^ Five clinical practice guidelines were solely developed by European medical societies: one from Britain, one from the UK, two from Spain, and one being a multi-disciplinary consensus among several medical organizations in Poland.^4,6,8,10,11^ Four clinical practice guidelines were from North American organizations, while one was from a Japanese organization.^3,5,9,13–16^

**Table 1.** General characteristics of included clinical practice guidelines; ACR = American College of Radiology; BTA = British Thyroid Association; NCCN = National Comprehensive Cancer Network; UKNMG = United Kingdom National Multidisciplinary Guidelines; AACE, ACE, AME = American Association of Clinical Endocrinologists, American College of Endocrinology, and Associazione Medici Endocrinologi; FESEO = Federacion de Sociedades Espanolas de Oncologia; PNS = Polish National Societies; ESMO = European Society for Medical Oncology; SEOM = Spanish Society of Medical Oncology; JAES = Japan Association of Endocrine Surgeons; AAES = American Association of Endocrine Surgeons; ATA = American Thyroid Association

Organisation	Journal	Year of Publication	Country /Region	Development method	Developers	Target User	# of references	Funding
ACR	*Oral Oncology*	2014	United States of America	Expert consensus via Modified Delphi study, literature review	Radiologists	Thyroid Specialists	100	—
BTA	Published on Website	2014	United Kingdom	Expert consensus, literature review	Radiologists, endocrine surgeons, endocrinologists, otolaryngologists, pathologists, surgical oncologists, medical oncologists, nurses, patient-led organisations	Thyroid Specialists	54	British Thyroid Association
NCCN	*Journal of National Comprehensive Cancer Network*	2015	United States of America	Expert consensus	Radiologists, endocrine surgeons, endocrinologists, otolaryngologists, pathologists, surgical oncologists, medical oncologists	Thyroid Specialists	103	—
UKNMG	*Journal of Laryngology & Otology*	2016	United Kingdom	Expert consensus	Data analysts and endocrine surgeons	Head and Neck Cancer Specialists	9	—
AACE, ACE, AME	*Endocrine Practice*	2016	International	Expert consensus, literature review	Endocrinologists, endocrine surgeons, oncologists	Thyroid Specialists	367	None
FESEO	*Clinical Translational Oncology*	2017	Spain	Expert consensus, systematic literature review	Medical oncologists and endocrinologists	Thyroid Specialists	60	—
PNS	*Endokrynologia Polska*	2018	Poland	Expert consensus, literature review	Endocrinologists, oncologists, paediatric endocrinologists, pathologists, surgical oncologists, anatomists, geneticists, reconstructive surgeons, endocrine surgeons, nuclear medicine specialists	Thyroid Specialists	102	—
ESMO	*Annals of Oncology*	2019	International	Expert consensus, literature review	Oncologists, nuclear medicine, pathologists, radiologists, head and neck surgeons	Thyroid Oncologists	166	ESMO
SEOM	*Clinical and Translational Oncology*	2020	Spain	Expert consensus	Oncologists	Thyroid Specialists	43	—
JAES	*Endocrine Journal*	2020	Japan	Expert consensus, systematic literature review	Surgeons, radiologists, endocrinologists, pathologists, supervisors, adviser	Thyroid Specialists	292	None
AAES	*Annals of Surgery*	2020	United States of America	Expert consensus, systematic literature review	Endocrine surgeons, surgical oncologists, pathologists, endocrinologists, otolaryngologists	Thyroid Specialists	1066	None
ATA	*Thyroid*	2021	United States of America	Expert consensus, systematic literature review	Medical oncologists, radiation oncologists, endocrinologists, molecular biologists, pathologists, otolaryngologists, endocrine surgeons, bioethicists, patient advocate stakeholders	Thyroid Specialists	330	None

One clinical practice guideline was developed using the modified Delphi study methodology, five were developed using only expert consensus, while six were developed using both expert consensus and a literature review. The developing bodies for each clinical practice guideline varied slightly but all included endocrine surgeons, otolaryngologists, endocrinologists, radiologists, and nuclear medicine specialists, with some clinical practice guidelines including patient-led organizations and/or experts of diverse personal background. The audience for the clinical practice guidelines was primarily healthcare providers, with some more specifically focusing on endocrine surgeons and otolaryngologists. All but three of the clinical practice guidelines provided funding sources.

### Guideline quality appraisal

Table 2 showcases the scaled-domain scores for the six Appraisal of Guidelines for Research and Evaluation II domains. The domain with the highest mean score was Domain 4 “clarity of presentation” (69.44 ± 16.75), followed by Domain 1 “scope and purpose” (68.87 ± 20.88). The domains with the lowest mean scores were Domain 5 “applicability” (7.12 ± 6.17) and Domain 3 “rigor of development” (50.26 ± 20.77). Domain 6 “editorial independence” had the greatest variability with a standard deviation of 31.8, while Domain 5 “applicability” had the least variability with a standard deviation of 6.2.

**Table 2.** Quality appraisal of included clinical practice guidelines using scaled domain scores

Agree II Domain	Intraclass Correlation Coefficient (ICC)	95% Confidence Interval	ICC Reliability
Scope and purpose	0.924	0.82 to 0.94	Excellent
Stakeholder involvement	0.873	0.80 to 0.97	Excellent
Rigor of development	0.914	0.83 to 0.95	Excellent
Clarity of Presentation	0.863	0.77 to 0.98	Excellent
Applicability	0.689	0.44 to 0.85	Good
Editorial independence	0.881	0.72 to 0.99	Excellent

Of the 12 clinical practice guidelines evaluated, only the 2014 British Thyroid Association and 2021 American Thyroid Association clinical practice guidelines were considered “high quality” with more than four domains having a score over 60.^4,15,16^ Four of the clinical practice guidelines were considered “average” with three or four domains having a score over 60.^7,10,13,14^ The remaining six clinical practice guidelines were rated as “low quality” with two or fewer domains receiving a score over 60.^3,5,6,8,9,11,12^

### Intraclass reliability

To identify the degree of consistency among the four reviewers (JK, NK, NS, ED) and evaluate the inter-rater reliability for the six domains appraised with the Appraisal of Guidelines for Research and Evaluation II tool, intraclass correlations were performed (Table 3). Of the six domains, five received an “excellent” intraclass reliability, with only the “applicability” domain (Domain 5) receiving a “good” intraclass reliability.

**Table 3.** Intraclass correlation coefficients for Appraisal of Guidelines for Research and Evaluation (AGREE) II domains; ACR = American College of Radiology; BTA = British Thyroid Association; NCCN = National Comprehensive Cancer Network; UKNMG = United Kingdom National Multidisciplinary Guidelines; AACE = American Association of Clinical Endocrinologists; FESEO = Federacion de Sociedades Espanolas de Oncologia; PNS = Polish National Societies; ESMO = European Society for Medical Oncology; SEOM = Spanish Society of Medical Oncology; JAES = Japan Association of Endocrine Surgeons; AAES = American Association of Endocrine Surgeons; ATA = American Thyroid Association

	Domain 1	Domain 2	Domain 3	Domain 4	Domain 5	Domain 6		
Organisation	Scope and purpose (%)	Stakeholder involvement (%)	Rigor of Development (%)	Clarity and Presentation (%)	Applicability (%)	Editorial Independence (%)	Overall Score (Average)	Overall quality
ACR	50.0	31.9	27.1	43.1	5.2	12.5	28.3	Low
BTA	86.1	86.1	68.2	81.9	17.7	75.0	69.2	High
NCCN	43.1	45.8	37.0	54.2	2.1	50.0	38.7	Low
UKNMG	37.5	15.3	13.0	72.2	3.1	0.0	23.5	Low
AACE	66.7	54.2	67.7	61.1	9.4	56.3	52.5	Average
FESEO	76.4	52.8	42.7	51.4	0.0	77.1	50.1	Low
PNS	65.3	51.4	68.2	90.3	3.1	68.8	57.8	Average
SEOM	83.3	40.3	34.4	80.6	2.1	56.3	49.5	Low
ESMO	44.4	48.6	37.5	70.8	8.3	66.7	46.1	Low
JAES	83.3	56.9	75.0	90.3	6.3	100.0	68.6	Average
AAES	98.6	62.5	58.9	51.4	8.3	97.9	62.9	Average
ATA	91.7	97.2	73.4	86.1	19.8	100.0	78.0	High
Mean ± SD	68.87 ± 20.9	53.59 ± 21.9	50.26 ± 20.8	69.44 ± 16.8	7.12 ± 6.2	63.37 ± 31.8		

## Discussion

Anaplastic thyroid carcinoma continues to be the most aggressive tumor of the thyroid and requires rapid diagnosis, treatment, and management to reduce its nearly 100 per cent mortality rate. With several treatment options available for anaplastic thyroid carcinoma, such as surgical debulking, external beam radiation, and chemotherapy, proper care necessitates a multi-disciplinary approach. As such, several clinical practice guidelines have been developed to identify best practices for the care of patients with anaplastic thyroid carcinoma.

- Anaplastic thyroid cancer is a rare but rapidly progressive cancer with poor prognosis
- Several organizations (American Thyroid Association, British Thyroid Association, etc.) have created clinical practice guidelines for the treatment and management of anaplastic thyroid carcinoma
- There are no studies known to us that evaluate the effectiveness of these clinical practice guidelines
- Our paper appraised the quality of clinical practice guidelines for treatment and management of anaplastic thyroid carcinoma
- Most treatment guidelines for anaplastic thyroid carcinoma were found to be low quality and varied from one another
- Standardization of practice guidelines with a larger focus placed on how to apply recommended practices is greatly needed

Clinical practice guidelines have been shown repeatedly to improve the level of medical care across several medical fields by reducing medical errors and improving patient outcomes.^22–24^ There has been an increased emphasis and utilization of clinical practice guidelines to guide clinical practice over the past several years.^23,24^ However, improvements in patient care can only occur if clinical practice guidelines are held to a high standard. The Appraisal of Guidelines for Research and Evaluation II tool was designed to appraise the quality of clinical practice guidelines and quantify the variability among these guidelines.^25^ Thus, our study aimed to evaluate the quality and applicability of the clinical practice guidelines for the treatment and management of anaplastic thyroid carcinoma using this tool.

Of the 12 appraised guidelines, only two were considered “high quality” based on the aforementioned criteria. The 2021 American Thyroid Association guidelines had the highest overall score, closely followed by the 2014 British Thyroid Association guidelines.^4,15,16^ Both of these had high scores in all six Appraisal of Guidelines for Research and Evaluation II domains except for the “applicability” domain. These clinical practice guidelines had experts across their respective countries from several medical disciplines such as otolaryngology, radiology, endocrinology, and oncology. Furthermore, they also included the perspectives of patient-led organizations.

The treatment of anaplastic thyroid carcinoma entails either surgical debulking with external beam radiation plus or minus chemotherapy, or external beam radiation plus or minus chemotherapy. Additionally, due to the high mortality rate and poor prognosis of this cancer, palliative and/or hospice care is also provided to patients. With several treatment options available, clinical practice guidelines must clearly identify their key recommendations. Our analysis showed that the domain with the highest overall rating was the “clarity of presentation” domain, which indicates the clinical practice guidelines clearly outlined their key recommendations and presented possible alternatives.

“Scope and purpose” was the domain with the second highest overall score. It evaluates whether the guidelines specifically described their objectives, health questions, and the populations to whom they were referring. Our study found that all but the 2014 American College of Radiology, 2016 United Kingdom National Multidisciplinary Guidelines, and 2019 European Society for Medical Oncology clearly identified the scope and purpose of their clinical practice guidelines^3,6,12^

The “rigor of development” domain has been shown to have the greatest influence on clinical practice guideline quality, with 8 of the 23 criteria within its domain.^26^ This domain focuses on the way in which evidence is gathered and synthesized into the formulated recommendations. Only 5 of the 12 clinical practice guidelines received a high rating in this domain. Aside from these five clinical practice guidelines, the other ones failed to explicitly state the relationship between the evidence and their final recommendations.

The “applicability” domain had the lowest overall rating of the six domains. This domain focuses on the barriers to implementation of the key recommendations and the resource implications of the guidelines.^17^ With the poor outcomes associated with anaplastic thyroid carcinoma, it is imperative that proper treatment be administered immediately, which can only be done if the barriers to implementation and required resources are adequately addressed. For clinical practice guidelines to receive a high rating in this domain, they must clearly address how relevant patient populations will be cared for and how the necessary resources, such as equipment, infrastructure, and personnel, will be acquired. Our results showed that this domain had the lowest overall score, with all of the guidelines receiving a low rating in this category.

### Limitations

As with all systematic reviews, our study has several limitations. The literature review only included clinical practice guidelines written in English, thus potentially limiting the international scope of our review. The literature search was also limited to specific databases outlines above such as MEDLINE/PubMed, Scopus, Embase, Cochrane, and Google Scholar. As such, guidelines included elsewhere were not considered in this study. The scientific accuracy of the guidelines was not addressed as the Appraisal of Guidelines for Research and Evaluation II tool was designed to gauge methodologic rigor of guidelines, not the scientific validity. Additionally, the grading of clinical practice guidelines via the Appraisal of Guidelines for Research and Evaluation II is subject to bias and subjectivity; however, to limit this, grading was performed by four independent reviewers who received standard, required training. To determine the grading reliability among the reviewers, intraclass correlations were calculated which found that five of the domains had “excellent” reliability with only one having “good” reliability.

### Recommendations

The key recommendations for treatment and management of anaplastic thyroid carcinoma were conglomerated from the 12 reviewed guidelines and are summarized below.

Initial evaluation of anaplastic thyroid carcinoma classically begins with FNA; however, core needle biopsy is shown to have a higher sensitivity and specificity for diagnosis. If the diagnosis of the thyroid mass is still inconclusive, an incisional biopsy may be performed. Before treatment, an established diagnosis of anaplastic thyroid carcinoma should be made. Molecular profiling should also be performed to assess for BRAF V600E mutations.

Alongside cytopathological diagnosis, CT scans with contrast should be taken of the neck, chest, abdomen, and pelvis along with MRI of the brain and positron emittance testing with CT to determine extent of tumour invasion, lymph node involvement, and presence of metastases. Furthermore, endoscopic evaluation of the vocal cords should be performed to determine invasion of the larynx. Additionally, multidisciplinary input should be attained regarding the patient's goals of care. Consultations with palliative care and/or hospice care should be provided prior to treatment to identify the patient's needs and how to best address them.

If R0/R1 resection is anticipated, most guidelines strongly recommend resection via a total thyroidectomy with or without a neck dissection. However, a radical resection is not recommended because of the poor prognosis of anaplastic thyroid carcinoma and the availability of adjuvant targeted therapies. For patients wanting an aggressive approach after resection, standard fractionation of intensity-modulated radiotherapy (RT) may be offered alongside concurrent therapy. If a patient has good performance status without metastases and the resection is R2 or the tumor is unresectable, then standard fractionation intensity-modulated RT with systemic therapy may be initiated.

For patients treated with radiation, adjuvant systemic therapy may be used. Most commonly, cytotoxic chemotherapy with a taxane (paclitaxel or docetaxel) with or without an anthracycline (doxorubicin) or platin (cisplatin or carboplatin) is recommended. If the patient is BRAF V600E positive, then combined BRAF/MEK inhibitors, such as dabrafenib/trametinib, can be considered. In stage 4C anaplastic thyroid carcinoma with high PD-ligand 1 expression, PDL1/PD1 inhibitors can be considered if no other targetable alterations exist or can supplement standard chemotherapy regimens via a clinical trial. In metastatic anaplastic thyroid carcinoma without clinical trial options, the aforementioned standard chemotherapy regimen may be used.

If patients present with neurological symptoms secondary to brain compression or metastases, daily dexamethasone should be administered alongside consultation of neurosurgery and/or radiation oncology services, if available. In the presence of bone metastases, intravenous bisphosphonate infusions, or subcutaneous RANKL-inhibitor injections should be administered. If there are symptomatic or threatening bone metastases, without structural compromise or threat to the spinal cord, palliative radiotherapy is recommended. However, if bone metastases present with structural compromise to weight-bearing regions or threaten spinal cord compression, then orthopedic fixation is recommended prior to palliative radiotherapy to improve quality of life.

## Conclusion

Anaplastic thyroid carcinoma is a rare, aggressive cancer with a poor prognosis, thus requiring immediate diagnosis and treatment. Several medical organizations across the world have put out clinical practice guidelines, consensus statements, and recommendations regarding the treatment and management of anaplastic thyroid carcinoma. With the high mortality associated with anaplastic thyroid carcinoma, it is imperative that each of these organizations utilize strong scientific evidence and provide standardized recommendations. Using the Appraisal of Guidelines for Research and Evaluation II tool, our results showed wide variability in the quality of the clinical practice guidelines published for the treatment and management of anaplastic thyroid carcinoma. Furthermore, only two clinical practice guidelines were identified as “high quality,” with half of the clinical practice guidelines being identified as “low quality” based on the criteria of the Appraisal of Guidelines for Research and Evaluation II instrument. Our findings indicate there are several areas of improvement for the standardization of practice guidelines, most specifically in the “applicability” and “rigor of development” domains.
